# Supplementary material for: Vps54 regulates Drosophila neuromuscular junction development and interacts genetically with Rab7 to control composition of the postsynaptic density
Source: Biol Open. 2020 Aug 25;9(8):bio053421. doi: 10.1242/bio.053421 (PMC7473652; doi:10.1242/bio.053421)
Supplement: Supplementary information [file biolopen-9-053421-s1.pdf]

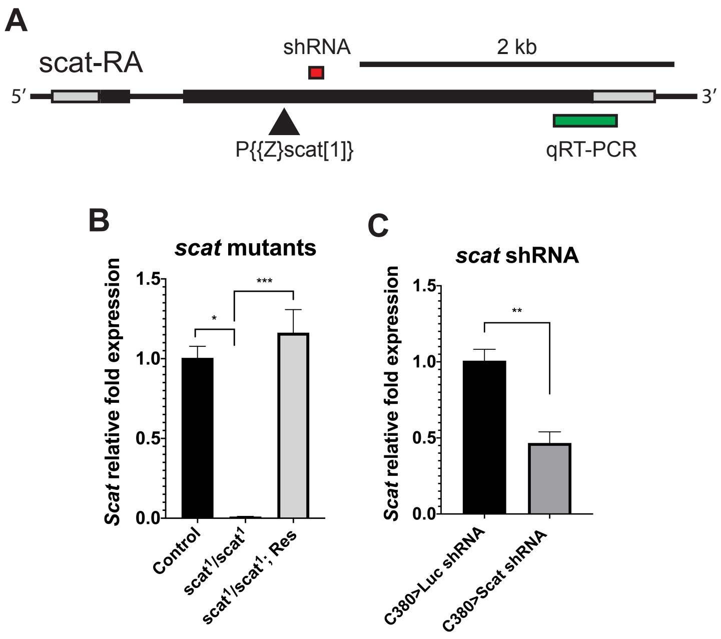

Fig. S1. The *scat[1]* allele. (A) This is a diagram showing the structure of the *scat* gene in *Drosophila*. Exons are indicated by boxes and the single intron indicated by a line. Gray boxes at the 3' and 5' ends indicate the untranslated regions (UTRs) and black boxes indicate coding sequence. The triangle shows the location of the P-element insertion in the second exon of the *scat* locus. Using an antibody targeting the N-terminal 200 amino acids of the Scat protein, it has previously been shown that this is a null allele (Fari et al., 2016). The region targeted by the *UAS-TRiP(HMS01910)* shRNA line is indicated by the red box. The green box at the 3' end indicates the location of the amplicon analyzed for qRT-PCR. (B) Results of qRT-PCR analysis of *scat* mRNA levels in the larval CNS of controls, *scat[1]* homozygotes, and the *scat[1]/scat[1]; scat-HA:scat/scat-HA:scat* rescue (Res) lines. *scat* mRNA is reduced in *scat[1]* homozygotes and restored to near control levels in the rescue line. (C) Results of qRT-PCR analysis of *scat* mRNA levels in control (*C380>Luc shRNA*) and *scat* (*C380>scat shRNA*) larval ventral ganglia. Levels of *scat* mRNA are reduced by at least 50%. Note that this is likely an underrepresentation because *C380-Gal4* drives expression in only a subset of neurons in the ventral ganglion. In both B and C, levels of *scat* transcripts were determined as described in the Methods and then normalized to the control. $N = 7$ ventral ganglia for each genotype. qPCR data shown in B was analyzed by one-way ANOVA followed by a Holm-Sidak multiple comparison test. qPCR data shown in C was analyzed by a two-way Student's t-test. Data are represented as the mean ± SEM for three technical replicates. * $p < 0.05$, ** $p < 0.01$, *** $p < 0.001$.

Biology Open • Supplementary information

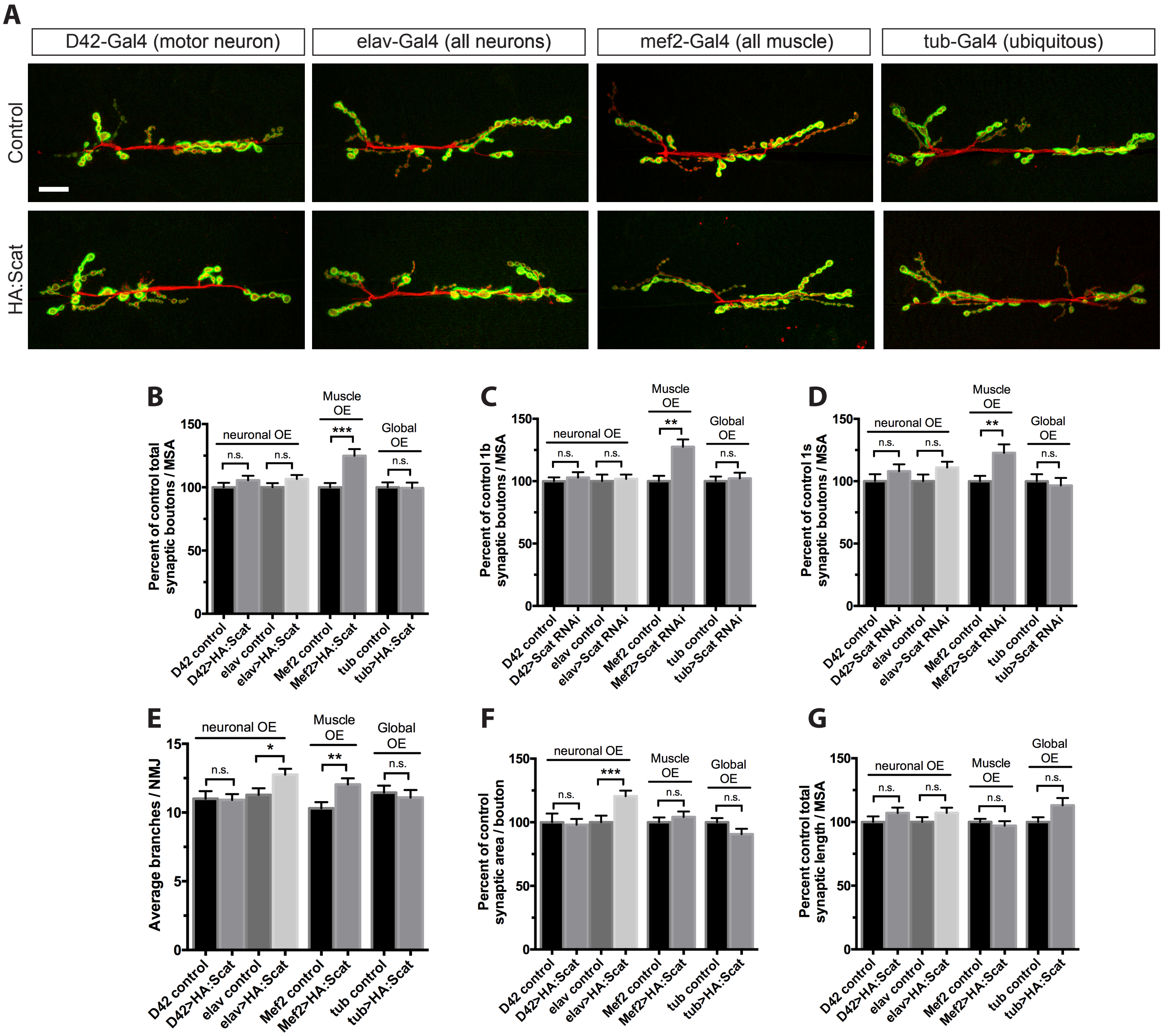

Fig. S2. Overexpression of *scat* has an effect on NMJ development. (A) The inducible *UAS-HA:scat* transgene was expressed using a motor neuron-specific driver (*D42-Gal4*), a strong pan-neuronal driver (*elav-Gal4*), a strong muscle-specific driver (*Mef2-Gal4*), or ubiquitously (*tubulin-Gal4*). NMJs at m6/7 in body segment A3 have been stained with antibodies targeting Dlg (green) and HRP (red). Images show maximum Z-projections. Scale bar, 20 μm. Quantification of the (B) total number of boutons, (C) type 1b boutons, and (D) type 1s boutons shows that bouton number is increased when HA:Scat is overexpressed in larval muscle but not when expressed ubiquitously or in neurons. (E) The number of branches is significantly increased when HA:Scat is strongly expressed in either neurons or muscle. For B-E, *N* = 22, 23, 19, 24, 30, 30, 23, and 23 in the order shown in graphs. (F) Total synaptic area is increased when strongly expressed in muscle. (G)

Biology Open • Supplementary information

There is no impact on total synaptic length. Synaptic area and length where quantified using the Morphometrics algorithm. For F and G, *N* = 22, 23, 19, 24, 28, 30, 24, and 27 in the order shown in graphs. All statistical analysis was done by Kruskal-Wallis followed by a Dunn's multiple comparison test. Unless otherwise indicated, all statistical comparisons shown have been compared to driver-specific controls (*driver/*+ heterozygotes). * $p < 0.05$, ** $p < 0.01$, *** $p < 0.001$.

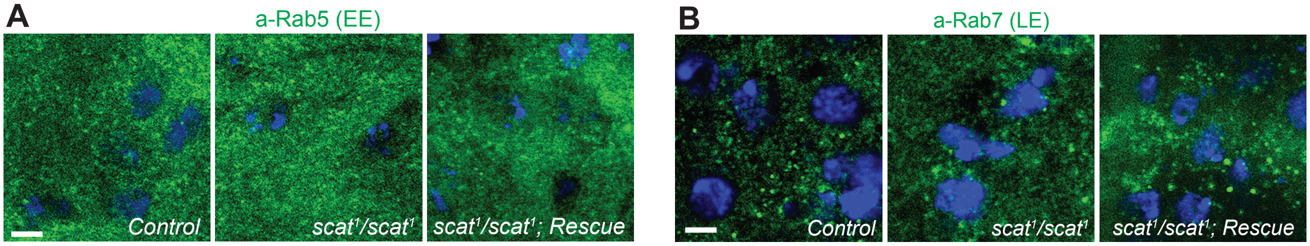

Fig. S3. *scat* mutants do not have defects in Rab5- or Rab7-positive endosomes. Using polyclonal antibodies targeting Rab5 and Rab7 (Hirata et al., 2015), there is no visible defect in the size, number, or distribution of EE or LE in *scat*^{1} mutant MNs. Images shown are single focal planes. Ventral ganglia from wandering third instar larvae from controls, *scat*^{1} homozygotes, and the *scat*^{1}*/scat*^{1}*; scat-HA:scat/scat-HA:scat* rescue lines were stained with antibodies targeting (A) Rab5 or (B) Rab7 (both green). Nuclei are marked by DAPI (blue). Scale bar, 2.5 μm.

Biology Open • Supplementary information

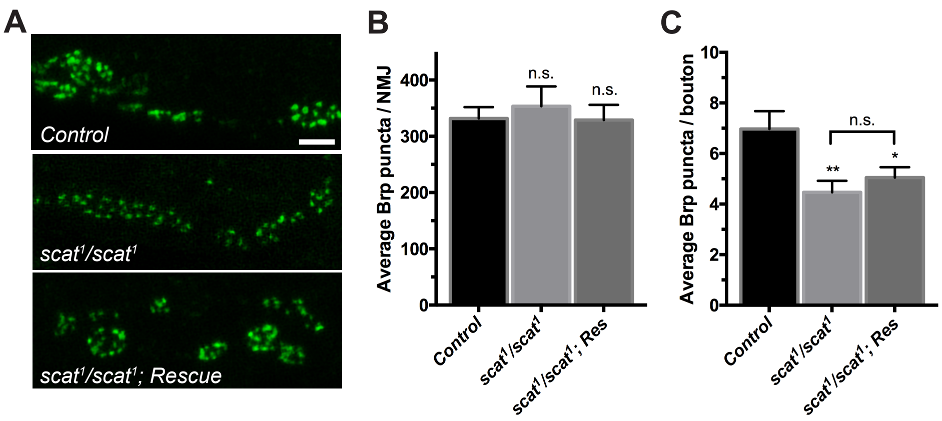

Fig. S4. *scat* mutants do not have active zone defects. A) NMJs innervating muscle 6/7 in body segment A3 from the indicated genotypes stained with an antibody targeting Brp (green). *scat* mutants have an increased number of small boutons with fewer Brp-positive puncta. Scale bar, 5 μm. B) The total number of Brp-positive punctae per NMJ does not change C) The number of Brp-positive punctae per synaptic bouton increases in *scat* mutants. *N* = 10 for each genotype. Statistical analysis was done using a one-way ANOVA followed by a Holm-Sidak multiple comparison test. Data in graphs are represented by the mean ± SEM.

Biology Open • Supplementary information

Table S1.

Primer list:

**Cloning of *scat* from LD2244 into pUAST (to generate the inducible HA:Scat line)**	
Primer pair	Sequence (5' to 3')
Scat_HAf	GATCTTGCGGCCGCATGGCC ACGACAAGATCCGC
Scat_HAr	GGTACCCTCGAGTTAAGCGTAATCTGGAACATCGTA TGGGTAGTAGAGCCAGATCTCCTCCA
**Cloning of *scat* from genomic DNA into pCASPR (to generate the genomic rescue line)**	
Primer pair	Sequence (5' to 3')
Scat rescue F1	GATCTTGCGGCCGCGTCAGCTGATTTTGCTCAGA
Scat rescue R1	TCAAGCGTAATCTGGAACATCGTATGGGTAGTAGAGCCAGATCTCCTCCA
Scat rescue F2	TACCCATACGATGTTCCAGATTACGCTTGAGCATTCCGAAGTATTCCATA
Scat rescue R2	GGTACCCTCGAGCTTTCACCCCTAAGTTGCAT
**Quantitative RT-PCR experiments (for Fig. S1)**	
Primer pair	Sequence (5' to 3')
Scat_qPf	ACGAAACTATCGAGCGGGAC
Scat_qPr	TTGAGCTGCCAATCTCTCGG
RpS3_qPf	TCTTTCTTTTCTGCGCACCA
RpS3_qPr	TCGCATTCATTTTGACGTCG

Biology Open • Supplementary information
